# Physiological and gene expression changes of *Cryptomeria fortunei* Hooibrenk families under heat stress

**DOI:** 10.3389/fpls.2023.1083847

**Published:** 2023-01-30

**Authors:** Jinyu Xue, Pingsheng Zeng, Jiebing Cui, Yingting Zhang, Junjie Yang, Lijuan Zhu, Hailiang Hu, Jin Xu

**Affiliations:** ^1^ Key Laboratory of Forest Genetics and Biotechnology of Ministry of Education, Co-innovation Center for Sustainable Forestry in Southern China, College of Forestry, Nanjing Forestry University, Nanjing, China; ^2^ Experimental Center of Subtropical Forestry, Chinese Academy of Forestry, Fenyi, China

**Keywords:** *Cryptomeria fortunei* Hooibrenk, heat stress, physiological analysis, plant cell ultrastructure, gene expression

## Abstract

Heat stress is one of the major abiotic stresses affecting plant growth and productivity. *Cryptomeria fortunei* (Chinese cedar) is an excellent timber and landscaping tree species in southern China thanks to its beautiful appearance, straight texture and ability to purify the air and improve the environment. In this study, we first screened 8 excellent *C. fortunei* families (#12, #21, #37, #38, #45, #46, #48, #54) in a second generation seed orchard. We then analyzed the electrolyte leakage (EL) and lethal temperature at 50% (LT_50_) values under heat stress, to identify the families with the best heat resistance (#48) and the lowest heat resistance (#45) and determine the physiological and morphological response of different threshold-resistance of *C*. *fortune* to heat stress. The relative conductivity of the *C. fortunei* families showed an increasing trend with increasing temperature, following an “S” curve, and the half-lethal temperature ranges between 39°C and 43.2°C. The activities of SOD and POD fluctuated in the early stage of stress but decreased after 37°C. We observed the changes in the cell ultrastructure at 43°C, and the mesophyll cell structure of #48 was less damaged than that of #45. Eight heat resistance gene, including *CfAPX1*, *CfAPX2*, *CfHSP11*, *CfHSP21*, *CfHSP70*, *CfHSFA1a*, *CfHSFB2a* and *CfHSFB4*, were all up-regulated in #45 and #48, and there were significant differences between #45 and #48 under different heat stress treatments. We found a significant difference in heat tolerance between #45 and #48, such that #48 shows higher heat tolerance capability and could be exploited in breeding programs. We conclude that the strongly heat-resistant family had a more stable physiological state and a wider range of heat stress adaptations.

## 1 Introduction

With the globe becoming warm, high temperature stress has become one of the major environmental stresses limiting plant growth, metabolism and productivity (Berry and Bjorkman, 1980; Hasanuzzaman et al., 2013). In recent years, the frequent occurrence of extremely high temperatures has posed a severe challenge to the ability of plants to withstand high temperatures (Borrell et al., 2020). In the published research on the effects of heat stress on plant physiology, there have been many studies on the relationship between heat tolerance and plant physiological indicators, but the types of plants studied have mainly included herbs and vegetables. In contrast, research on forest trees has been limited. Different plants have different heat-resistance mechanisms, and the same plant may also be affected by the synergistic effect of multiple heat-resistance mechanisms (Hasanuzzaman et al., 2010). Compared with agricultural crops, forest trees have more complex cultivation conditions, more resilience mechanisms and longer growth cycles (Bi and Zhuge, 2008). During their growth and development, trees are affected by various environmental factors, among which temperature, light and water are the most important abiotic factors influencing tree growth. When the damage caused by high-temperature stress exceeds a plant’s own regulatory capacity, the plant exhibits heat-damage symptoms (Nahar et al., 2015).

Heat stress usually means that the temperature exceeds the threshold level for a period of time, which is enough to cause irreversible damage to plant growth and is a serious threat to global crop production (Vetter et al., 1958). The cell membrane is the critical barrier between cells and the external environment. Therefore, heat stress can initially cause changes in cell membrane permeability, leading to the denaturation of membrane proteins, separation of cell membrane lipids, and disruption of normal cell physiological activities (Hemantaranjan et al., 2014). Changes in cell membrane permeability will cause the cell membrane to lose its ability to select substances, the electrolytes in the cytoplasm to extravasate, and the conductivity of cell tissue fluids to increase (Jiang et al., 2016). Chlorophyll synthesis is sensitive to heat stress and is a good indicator of heat stress damage (Gosavi et al., 2014). Heat stress can cause various changes in chloroplasts, such as changes in thylakoid structure, the loss of granal stacking, and granal swelling (Ashraf and Hafeez, 2004; Rodríguez et al., 2005). In addition, heat stress-mediated changes in chloroplast ultrastructure can be used to indicate the heat tolerance of plant varieties at the cellular level (Zou et al., 2017).

The protective enzymes in plants mainly include catalase (CAT), peroxidase (POD), superoxide dismutase (SOD) and ascorbate peroxidase (APX) (Hong et al., 2021; Hu et al., 2021). For instance, there was a significant negative correlation between the activities of SOD and POD in different bottle gourd seedlings and the heat damage index of bottle gourds (Tian et al., 2020). SOD mainly scavenges superoxide anions in plants and converts them into H_2_O_2_ and O_2_, thereby maintaining cellular homeostasis and protecting cells from oxidative damage (Talbi et al., 2015). One of the important physiological mechanisms of plants in response to heat stress is the accumulation of osmoregulatory factors. The main osmotic regulators in plant cells mainly include proline, soluble sugars, and soluble proteins (Kaplan et al., 2004).

The survival strategy of plants under heat stress conditions often involves altering gene expression during transcription/translation, resulting in the production of heat shock proteins (HSPs). During cell growth and development, HSPs participate in protein synthesis, folding, and target protein degradation, thereby maintaining the stability of the cell membrane system (Usman et al., 2015; Khan and Shahwar, 2020). In plant genomes, approximately 7% of the coding sequences are assigned to transcription factors (TFs). Compared with animals and yeast, many of them belong to large gene families, such as heat stress transcription factors (HSFs) (Fragkostefanakis et al., 2015). Mishra found that in tomato (*Solanum lycopersicum*), at each developmental stage, the plants with *HsfA1a* silencing were more sensitive to heat stress than the wild type (Mishra et al., 2002).

*Cryptomeria fortunei* Hooibrenk is an evergreen tree species of the Cupressaceae family. It is an excellent timber and garden tree species in southern China (Zhang et al., 2021). With increasing global warming, the living environment of *C. fortunei* is also worsening. Therefore, it is of great theoretical and practical significance to study the physiological mechanism and genes related to the heat tolerance of *C. fortunei* in response to heat stress for subsequent molecular genetics and breeding research. In this study, we preselected the most and least thermo sensitive families among the eight tested families. We then analyzed the changes in needle phenotype, antioxidant activity, and chlorophyll content and ultrastructure of the two families under heat stress. Finally, we used the transcriptome data of *C. fortunei* available in our laboratory to identify heat resistance-related genes and explore their expression levels in families of *C. fortunei* with different levels of heat resistance, which laid a foundation for molecular breeding of heat resistance in *C. fortunei*.

## 2 Materials and methods

### 2.1 Plant materials

Our collection of *C. fortunei* is mainly derived from the eastern part of China (**Table 1**). The seedlings of eight elite *C. fortunei* families that were the offspring of the second orchard of *C. fortunei* in Xiapu forest farm, Xiapu County, Fujian Province, were selected. The material was harvested in 2018, and the seedlings were raised in 2019. In May 2021, we moved these to our laboratory in Nanjing (118°50’ E, 32°05’ N) for this study. To carry out heat stress, we pretreated 2-year-old healthy *C. fortunei* plants in the growth chamber under a 12/12-h photoperiod (day/night) at 25 °C/20 °C and 60% humidity and conducted a 4-week preliminary experiment.

**Table 1:** Materials and sources.

Families	Sources	Longitude (E)	Latitude (N)	Annual temperature range (◦C)	Average Annual Precipitation (mm)
#12	Shuimen Forestry Farm, Xiapu County, Fujian Province	120°06’	27.00’	17-23	1640
#21	Shiyang Forest Farm, Wencheng County, Zhejiang Province	119°50’	27°50’	16-25	1725
#37	Lushan Forest Farm, Jiujiang County, Jiangxi Province	116°05’	29°45’	11-18	1745
#38, #45, #46, #48	Xiapu Forest Farm, Xiapu County, Fujian Province	119°57’	26°52’	17-23	1640
#54	Tianmushan Forest Farm, Lin’an County, Zhejiang Province	119°25’	30°20’	13-22	1386

### 2.2 Heat stress treatment

The shoots with consistent growth across the families of *C. fortunei* were collected and subjected to heat stress treatment at 25°C, 29°C, 33°C, 37°C, 39°C, 41°C, and 43°C. Starting from 25°C, the first 3 temperatures were increased at 4°C/h, and the temperature was increased at 2°C/h after 37°C. When the target temperature was reached, the treatment was performed for 9 hours. Six needles from three positions were taken after 12 h of treatment. The plants of families #45 and #48 were sampled after 0 h, 3 h, 6 h and 9 h of heat stress treatment (43°C). The plants of groups #45 and #48 were sampled after 0h, 3 h, 6 h and 9 hof heat stress treatment (43°C). These samples were wrapped in tin foil, placed in liquid nitrogen for rapid freezing, and then stored in an ultralow temperature refrigerator at -80°C. The experimental treatment was repeated three times, and three plants were treated each time.

### 2.3 Electrolyte leakage (EL) and lethal temperature at 50% (LT_50_)

EL was measured according to the method of Zhang with some modifications (Zhang et al., 2020). Here, 0.2 g of the treated sample needles was weighed; samples were placed in 10 ml of distilled water and shaken with a shaker at room temperature. After 4 hours of incubation, the conductivity was measured by a conductivity meter (DDS-307, Leici Instruments Co., Shanghai, China). The formula EL(%)=(E_1_−E_0_)/(E_2_−E_0_)×100 was used, wherein the conductivity of distilled water was E_0_ and that of the extract was E_1_. The sample was heated in a boiling water bath for 15 min. After cooling to room temperature, the ultimate conductivity was measured as E_2_.

In combination with the logistic equation, the relative conductivity was fitted with the logistic regression equation as Y=K (/1+ae^-bt^), where Y is EL, t is temperature, K is the maximum EL, and a and b are unknown parameters. The equation was processed so that the second derivative was equal to zero, and the values of a and b and the correlation coefficient R were obtained. The inflection point temperature of the relative conductivity curve corresponding to the processing temperature is the half lethal temperature, that is, LT_50_=lna/b (Janáček and Prášil, 1991; Huang et al., 2016).

### 2.4 Chlorophyll content

Needle samples (0.2 g) of #45 and #48 after heat stress treatment were weighed and placed in a mortar; calcium carbonate, quartz sand and 2-3 ml of 95% alcohol were added to grind the sample into a homogenate, and 7-8 ml of 95% alcohol was added until the tissue turned white. The supernatant was centrifuged at 500 rpm 3 times at room temperature for 10 minutes each time, and the OD_665_ (optical density at 665 nm) and OD_649_ (optical density at 649 nm) of the supernatant were measured with a spectrophotometer. The chlorophyll (a+b) content was measured according to the method described by Lichtenthaler and Wellburn (Lichtenthaler and Wellburn, 1983).

### 2.5 Determination of SOD and POD activity

The needles of #45 and #48 were collected as samples after heat stress treatment. We then weighed the needles (0.2 g), ground them in liquid nitrogen, and suspended them in 3.0 ml of a solution consisting of 50 mM phosphate buffer pH 7.8. The homogenate was centrifuged at 10000 rpm for 20 minutes at 4°C. The supernatant was taken to analyze SOD and POD activities. SOD activity analysis was based on the ability of SOD to inhibit the photochemical reduction of nitroblue tetrazolium (NBT) (Luo et al., 1999). The activity of SOD was expressed as the activity units of the enzyme that inhibited 50% of the NBT. SOD activity was expressed as U g^-1^ FW protein. POD activity was measured by using guaiacol as the substrate (Thomas et al., 1982). One unit of POD activity was defined as a change of 0.01 in 1 min at 470 nm. POD activity was expressed as U g^-1^ FW min^-1^.

### 2.6 Ultrastructural observations

Needle samples # 45 and # 48 were collected and subjected to 37°C and 43°C for 24 hours, and samples at 25°C were collected as controls. After treatment, three mature needles were sampled. The needles were cut into a 3 mm segment size with a blade and placed in a 5 ml penicillin bottle; 2 ml of 3% glutaraldehyde solution was added, the air in the bottle was extracted with a syringe, which caused the needles to sink entirely to the bottom of the bottle. Samples were then stored in a 4°C refrigerator for 24 h. The specimens were rinsed with 0.1 mol L^-1^ phosphate buffer solution with a pH of 7.0 3 times for 30 minutes each time. The specimens were subsequently dehydratedin a series of graded alcohol solutions, namely, 15%, 30%, 50%, 70%, 80%, 90%, 95%, 100% solution, followed by acetone solution at room temperature. The samples were infiltrated and embedded with Poly Bed 812 epoxy resin, dried and stored for 8 hours. The samples were then sectioned using ultratome III ultramicrotome (LKB, Bromma, Stockholm, Sweden). The samples were subsequently stained in uranium dioxide acetate and lead citrate and then observed *via* the transmission electron microscope (TEM) (JEM-2100, JEOL Ltd., Tokyo, Japan). From the TEM images, ten chloroplasts were selected for observation. The shape of the chloroplast is approximated to be an ellipse, and the calculation formula of ellipse area is as follows: S=πab, where S is the elliptical area (µm^2^), a is the semimajor axis (µm), and b is the semiminor axis (µm).

### 2.7 Gene expression

The total RNA extracted from the needles of *C. fortunei* treated for 0, 3, 6, and 9 h under heat stress at 43°C was used as the template. A TaKaRa Mini BEST Plant RNA Extraction Kit was used to extract total RNA (Spin-column) (Takara, Beijing, China). The RNA concentration was measured by spectrophotometer (NanoDrop, 2000; Thermo Scientific, Waltham, Massachusetts, USA). Reverse transcription was performed with a HiScript III RT SuperMix for qPCR (+gDNA wiper) (Vazyme, Nanjing, China) reverse transcription kit according to the manufacturer’s instructions. According to the existing *C. fortunei* transcriptome sequencing data in our laboratory, eight genes related to heat resistance were screened. Homologous sequences were identified by the NCBI online tool BLAST (https://blast.ncbi.nlm.nih.gov/Blast.cgi ), and specific primers used were designed by Primer Premier 5.0. The CYP gene of *C. fortunei* was used as the internal reference gene. The reverse transcription cDNA template was diluted 10 times, and three replicates were evaluated. A ChamQ SYBR qPCR Master Mix (Low Rox Premixed) kit (Vazyme) was used for qRT-PCR analysis. All qPCRs were carried out on an Applied Biosystems 7500 real-time PCR system (ABI, Foster City, CA, USA). The expression levels were calculated using the 2^-ΔΔCt^ method (Zhang et al., 2021). **Table 2** lists all primers used to quantify the reverse transcription polymerase chain reaction (PCR). Experimental consumables, including 96 well 0.2 ml semi-skirt PCR plates, were obtained from NEST Biotechnology Co. Ltd (Wuxi, China).

**Table 2:** qRT-PCR primer sequence.

Primer name	Primer sequence(5’-3’)
*CfAPX1*-F	TGTGAGCACCAGACAAAGCC
*CfAPX1*-R	AGCCTGAGCCACCAGAAGAG
*CfAPX2*-F	CTCAAAGCCAGAGCGTTCCT
*CfAPX2*-R	CCGTTTGCCTGATGCTACTC
*CfHSP11*-F	ATCACCGAGTAGAACGCTCG
*CfHSP11*-R	GTATGGCTTCGGCTCCTGTT
*CfHSP21*-F	GATTTGGTTGCTCTCCAGGC
*CfHSP21*-R	AAGGACACCAGGGCAATGTT
*CfHSP70*-F	AAGCGGATCACTTTCAGGCA
*CfHSP70*-R	CAGAGCTGTTTTGGATGCCG
*CfHSFA1a*-F	CGTCAGCTGTCTGGTCAAGT
*CfHSFA1a*-R	AACGGCCCTCCTGTAATTGG
*CfHSFB2a*-F	CCGTGGAAAACAAAGCATCT
*CfHSFB2a*-R	AAGATCGTTGCATTGCTGTG
*CfHSFB4*-F	GGAAGTCTGCGATCGATGTT
*CfHSFB4*-R	TGACACGACTCAACCGCTAC
*CYP*-F	TCTCGGGCAGCATTTCACGC
*CYP*-R	AGCCGAAACTGGCGCCAACA

### 2.8 Statistical analysis

GraphPad Prism 8 was used for statistical analysis and mapping. When p ≤ 0.05, the Pearson rank correlation method was used to calculate the bivariate correlation between traits and corresponding gene expression levels. The data were analyzed by one-way ANOVA, and Duncan’s test was conducted in accordance with p ≤ 0.05. These analyses were conducted using SPSS 26.0.

## 3 Results

### 3.1 Individual *C. fortunei* families show varying degrees of heat resistance

**Figure 1:**
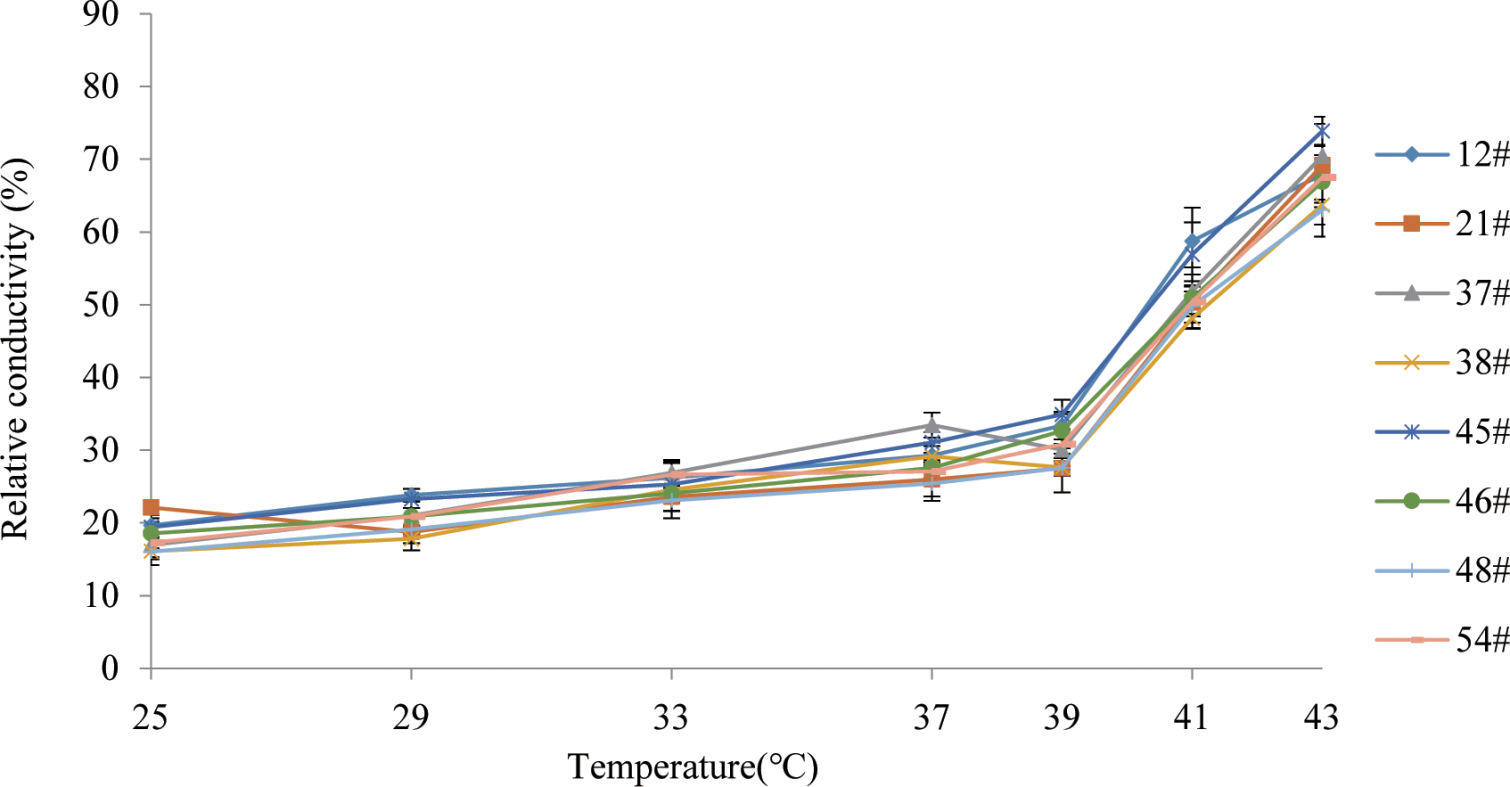
Variations of electrolyte leakage of eight *C. fortunei* families under heat stress.

We aimed to preselect the most and least heat resistant *C. fortunei* families from our set of 8 by analyzing the EL and LT_50_ values in response to heat stress. The EL values of all *C. fortunei* families increased gradually with increasing temperature, showing a consistent “S”-shaped curve response (**Figure 1**). We found that the lowest LT_50_ was for #45, which was 39.9°C, and the highest LT_50_ was for #48, which was 43.1°C (**Table 3**). Subsequently, we selected these two families for further physiological index tests and related experiments to study the widest possible range of heat-resistant responses within *C. fortunei*.

**Table 3:** Regression equation of electrolyte leakage and temperature and lethal temperature of 50% of *C. fortunei*.

Families	Equation parameters		Fitting Degree (R^2^)	Lethal temperature of 50% (°C)
	a	b		
#12	79.6	0.108	0.868	40.7
#21	68.9	0.099	0.791	42.6
#37	108.9	0.115	0.888	40.9
#38	100.5	0.108	0.893	42.8
#45	110.0	0.118	0.871	39.9
#46	85.2	0.106	0.877	41.9
#48	96.7	0.106	0.874	43.1
#54	89.8	0.107	0.874	41.9

### 3.2 Heat stress causes needle damage in *C. fortunei* families

**Figure 2:**
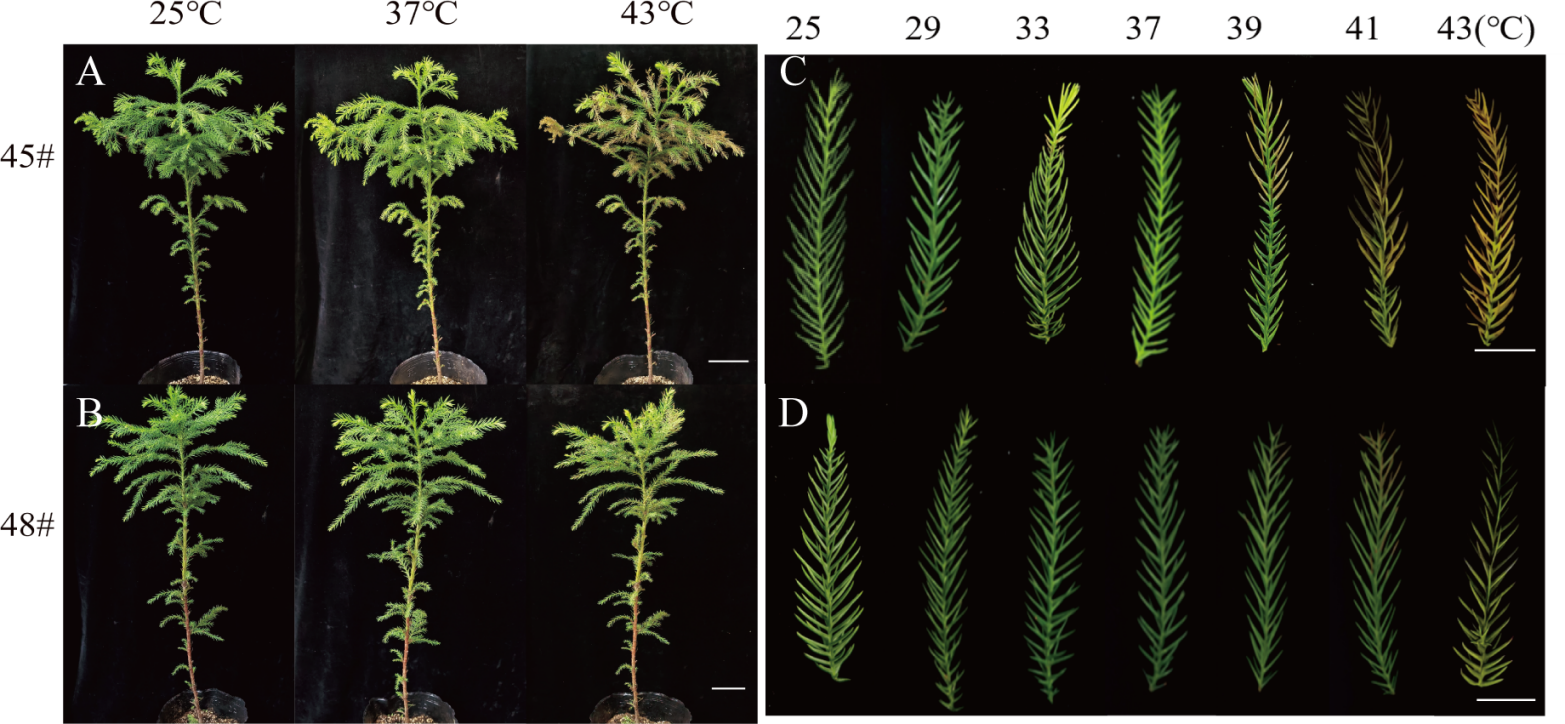
The phenotypes of the *C fortune* families changed significantly under heat stress. **(A)** #45 phenotype at 25°C, 37°C and 43°C; **(B)** #48 phenotype at 25 °C, 37 °C and 43 °C; **(C)** needle phenotype of #45; **(D)** needle phenotype of #48. **(A, B)** Bars=10 cm; **(C, D)** Bars=2 cm.

Heat stress often leads to changes in leaf color in plants. After heat stress treatment at 37°C, compared with the control (25°C), the needles of #45 and #48 showed no significant difference in needle shape or color (**Figures 2A, B**), yet at ≥ 39°C, they showed different degrees of etiolation. The degree of conifer browning damage increased significantly with increasing heat stress. After treatment at 39°C, the needles of #45 were browned and dried, and at 43°C, most of the needles had dried (**Figures 2C, D**) . Furthermore, heat stress also caused the top needles to wilt and droop, making them thicker and easier to detach. In addition, compared with the #45 family, phenotypic changes in the #48 family under high-temperature stress were not obvious, and the degree of needle browning and drying was lower. This is consistent with the fact that the LT_50_ of the #48 family is higher than that of the #45 family (**Table 3**).

### 3.3 Chlorophyll contents of the *C. fortunei* families change under different heat stresses

**Figure 3:**
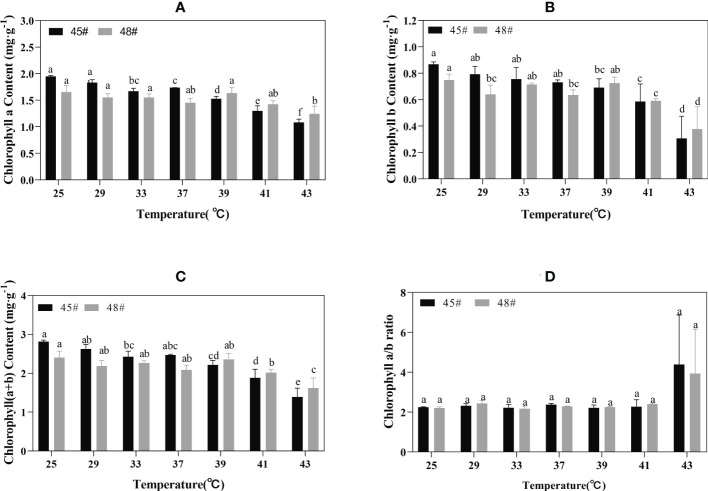
Chlorophyll changes in *C. fortune* families under heat stress. **(A)** Chlorophyll a content; **(B)** Chlorophyll b content; **(C)** Chlorophyll (a+b) content; **(D)** Chlorophyll a/b ratio. Each value in the graph is the mean ± standard error (n=3). The different patterns indicate different treatment temperatures, and the lowercase letters above each bar indicate significant differences (p < 0.05).

Chlorophyll synthesis is sensitive to heat stress and is a good indicator of heat stress injury (Gosavi et al., 2014). Heat stress had an adverse effect on the chlorophyll content of seedlings of the *C. fortunei* families. We found that the chlorophyll a content of the #45 and #48 needles showed a decreasing trend with increasing temperature. Under the 25°C-39°C treatment, the change was relatively small, and the decrease was obvious after treatment at 39°C (**Figure 3A**). The chlorophyll a, b and chlorophyll (a+b) contents of #48 needles were less than those of #45 needles following heat stress treatment under 39°C but more than those of #45 needles at 39-43°C (**Figures 3A–C**). These results indicated that #48 changed slowly under heat stress and was more stable under heat stress than #45. However, the chlorophyll a/b ratio did not increase (**Figure 3D**).

### 3.4 The antioxidant system of the *C. fortunei* families changes under different heat stresses

**Figure 4:**
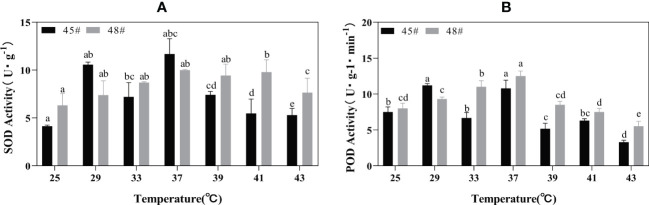
Changes in the POD and SOD contents of *C fortunei* families under heat stress. **(A)** SOD content; **(B)** POD content. Each value in the graph is the mean ± standard error (n=3). The different patterns indicate different treatment temperatures, and the lowercase letters above each bar indicate significant differences (p < 0.05).

Heat stress treatment significantly affected the activities of POD and SOD in #45 and #48. During heat stress, the SOD activities of #45 and #48 needles were the highest at 37°C; compared with 25°C, the increase was 182.61% and 158.22%, respectively (**Figure 4**). With increasing temperature stress, the POD activities of #45 and #48 showed a trend of first increasing and then decreasing, similar to the changes in SOD activities. The POD activity also showed a downward trend after 37°C, and #45 at 43°C decreased by 68.99% compared with that at 37°C. After treatment at 39°C, both the SOD and POD activities of #48 were higher than those of #45 (**Figure 4**).

### 3.5 Effect of heat stress on the ultrastructure of mesophyll cells of *C. fortunei*

**Figure 5:**
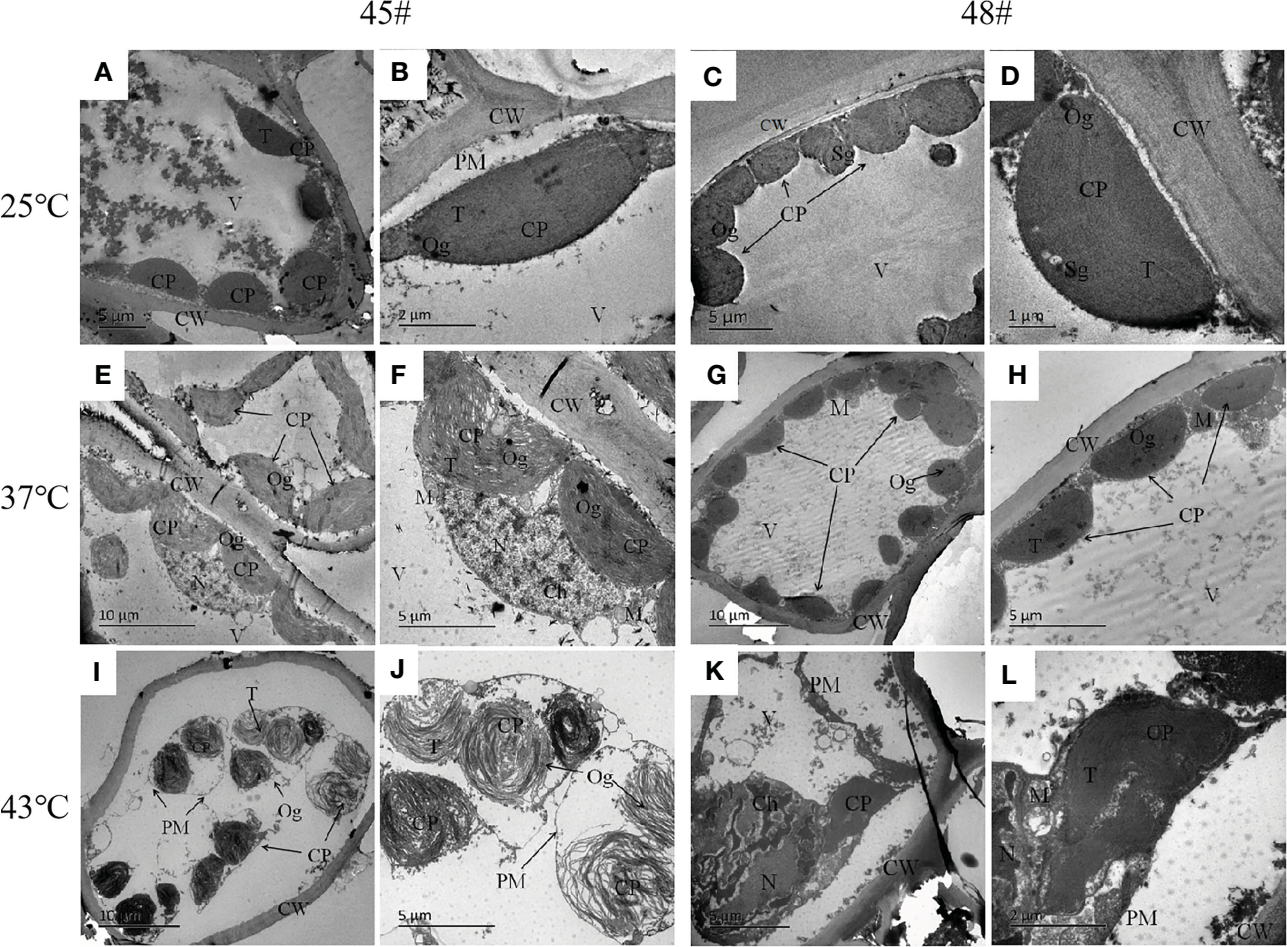
Changes in the ultrastructure of mesophyll cells in #45 and #48 under heat stress **(A, B)** #45 room-temperature treatment; **(C, D)** #48 room-temperature treatment; **(E, F)** #45 subjected to 37°C; **(G, H)** #48 subjected to 37°C; **(I, J)** #45 subjected to 43°C; **(K, L)** #48 subjected to 43°C. Cell wall, CW; Membrane, PM; Vacuole, V; Chloroplast: CP; Thylakoids, T; Starch granules, SG; Mitochondria, m; Nucleus, n; Chromatin, ch; Osmiophilic particles, Og.

Under heat stress, the ultrastructure of mesophyll cells of the #45 and #48 families changed obviously. Compared with the control (25°C) (**Figures 5A–D**), we found that the ultrastructure of #48 was not significantly different under heat stress at 37°C, but the number of osmiophilic granules in its chloroplasts was significantly increased, and no starch granules were observed (**Figures 5G, H**). In addition, we also found that #45 showed similar changes, but #45 had more obvious changes in chloroplast structure than #48, and it could be clearly seen that the thylakoid sheet structure in the chloroplast was distorted and loose (**Figures 5E, F**). Both #45 and #48 mesophyll cells exhibited severe plasmolysis at 43°C, and some organelles moved toward the center of the cell (**Figures 5I, K**). In the cells of #45, the tonoplast membrane was ruptured, the chloroplast structure was further expanded, deformed or even ruptured, and the internal osmiophilic granules increased. The thylakoid sheets were loose and disordered (**Figure 5J**). In contrast, the cells of #48 were less damaged. We observed a relatively intact plasma membrane, but a damaged nucleus and mitochondrial structure, in which chromatin within the nucleus aggregated into darker clumps, and the chloroplast structure was ruptured, but its thylakoid sheets remained tightly packed (**Figure 5L**). According to the above results, the ultrastructure of mesophyll cells in both families responded to heat stress, and the #45 family was more susceptible to heat stress damage than the #48 family.

### 3.6 Effect of heat stress on the chloroplast size of *C. fortunei*

The photosynthesis efficiency depends not only on the number of chloroplasts in cells but also on the surface area of chloroplasts (Bondada et al., 1994). The sectional area of #45 and #48 chloroplasts first increased and then decreased with increasing heat stress (**Table 4**). The changes in the chloroplast cross-sectional area of #45 at 37°C were significantly increased compared with the control (25°C), but the changes in #48 at 37°C were not significant. We found that the chloroplast cross-sectional area of #48 changed significantly when treated at 43°C, and the chloroplast cross-sectional area decreased significantly. We therefore conclude that the effect of heat stress on #45 was greater than that on #48, which showed weaker heat tolerance and more severe damage.

**Table 4:** Effects of heat stress on cross sections of chloroplasts in two families.

Temperature (°C)	#45			#48		
	Length of Long Axis (µm)	Length of Short Axis (µm)	Cross-Section Area of Chloroplast (µm^2^)	Length of Long Axis (µm)	Length of Short Axis (µm)	Cross-Section Area of Chloroplast (µm^2^)
25	6.46 ± 0.89b	3.75 ± 0.91b	18.63 ± 3.56b	5.67 ± 0.64b	3.71 ± 0.37b	16.51 ± 2.56ab
37	8.50 ± 0.71a	4.08 ± 0.45b	27.23 ± 3.35a	6.17 ± 0.84b	3.71 ± 0.55b	17.81 ± 2.86ab
43	5.96 ± 0.53b	5.08 ± 0.42a	23.90 ± 3.66a	6.34 ± 0.75b	2.79 ± 0.54c	14.09 ± 3.24c

a, b, and c indicate that the two families (#45 and #48) have significant differences in long axis, short axis, or chloroplast cross-sectional area under different heat stress (p < 0.05).

### 3.7 Expression of heat resistance genes in *C. fortunei* families under heat stress

**Figure 6:**
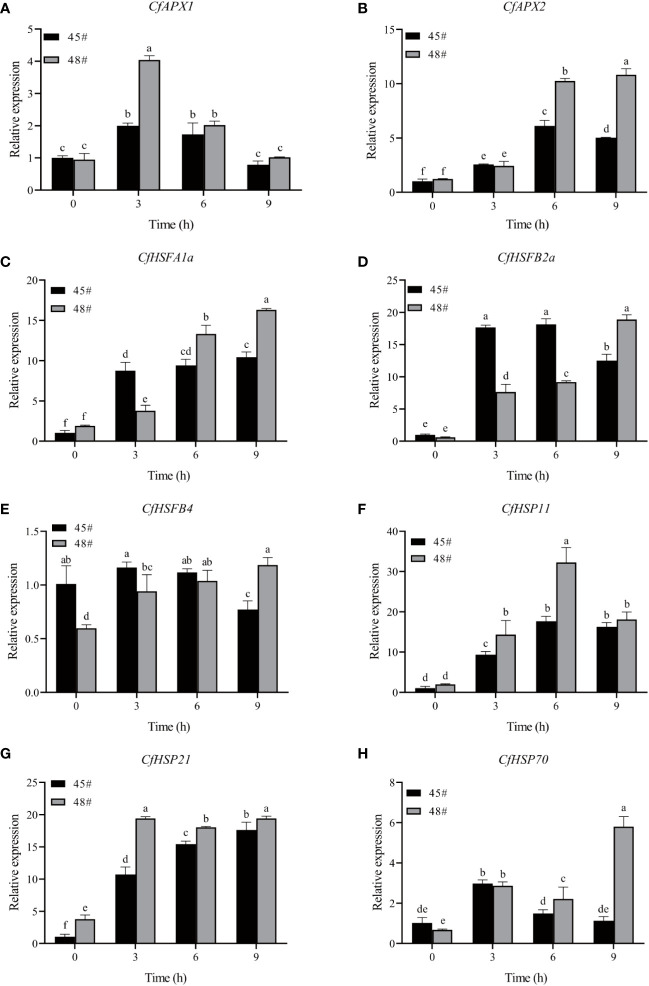
Effects of heat stress on gene expression in *C. fortunei* needles. The time course of heat in *C. fortune* shifted to 43°C. **(A)** *CfAPX1*; **(B)** *CfAPX2*; **(C)** *CfHSP11*; **(D)** *CfHSFB2a*; **(E)** *CfHSFB4*; **(F)** *CfHSP11*; **(G)** *CfHSP21*; **(H)** *CfHSP70*. Each value in the graph is the mean ± standard error (n=3).

To gain more insights into the response of *C. fortunei* to heat stress, we evaluated eight heat-resistance-related genes screened from the existing transcriptome data under 43°C culture conditions (**Figure 6**). We found that the transcript levels were significantly different in #45 and #48. The relative expression of *CfAPX1* under heat stress showed a trend of first increasing and then decreasing in both families, and its gene expression in #48 was higher than that in #45 (**Figure 6A**). Under heat stress, the relative expression of *CfAPX2* showed a trend of first increasing and then decreasing in #45 and reached the highest level at 6 h, which was 6.0 times that of the control (0 h) (**Figure 6B**). The expression level of *CfHSFB2a* in #45 was higher than that in #48 at 0 h, 3 h and 6 h (**Figure 6D**). The relative expression of *CfHSP70* in #45 reached the highest level at 3 h and decreased significantly thereafter. We observed that between the two families, the relative expression levels of genes in #48 and #45 were not significantly different under the 0h and 3 h treatments, while gene expression in #48 was significantly higher than that in #45 under the 6 h and 9 h treatments (**Figures 6F–H**).

### 3.8 Correlation of various physiological indicators in *C. fortunei* families under heat stress

At 25°C, the EL of the #45 family was significantly positively correlated with five physiological parameters, and there was a positive correlation between these five physiological parameters (**Table 5**). At room temperature, the EL of #48 had no significant correlation with chlorophyll a; however, chlorophyll (a+b) content and SOD activity were negatively correlated. In addition, POD in #48 was significantly positively correlated with EL but negatively correlated with the other four physiological indicators.

**Table 5:** Correlation of various physiological indicators in *C. fortunei* families under heat stress.

		#45					#48				
		Chla	Chlb	ChI(a+b)	SOD	POD	Chla	Chlb	ChI(a+b)	SOD	POD
25°C	EL	0.710*	0.710*	0.991**	0.912**	0.965**	-0.771*	-0.296	-0.473	-0.042	0.949**
	Chla		1.000**	0.797*	0.358	0.500		0.836**	0.925**	0.668*	-0.933**
	Chlb			0.797*	0.358	0.500			0.982**	0.967**	-0.583
	ChI(a+b)				0.849**	0.921**				0.900**	-0.727
	SOD					0.988**					-0.356
37°C	EL	-0.771*	-0.296	-0.473	-0.042	0.949**	-0.958**	-0.974**	-0.964**	0.919**	0.997**
	Chla		0.836**	0.925**	0.668*	-0.933**		0.998**	1.000**	-0.993**	-0.935**
	Chlb			0.982**	0.967**	-0.583			0.999**	-0.985**	-0.955**
	ChI(a+b)				0.900**	-0.727*				-0.991**	-0.942**
	SOD					-0.356					0.888**
43°C	EL	-0.033	-0.207	-0.161	0.964**	0.902**	-0.025	-0.949**	-0.623	-0.216	-0.351
	Chla		0.985**	0.992**	-0.297	0.401		0.364	0.798**	0.982**	-0.927**
	Chlb			0.999**	-0.459	0.235			0.852**	0.535	0.012
	ChI(a+b)				-0.417	0.280				0.898**	-0.514
	SOD					0.756*					-0.839**

*Indicates P < 0.05; ** Indicates P < 0.01.

Under 37 °C heat stress, the EL and POD of #45 and #48 were significantly positively correlated with each other and negatively correlated with the chlorophyll content (**Table 5**). In the #45 family, POD was negatively correlated with the other 4 physiological parameters. In addition, under 37 °C treatment, SOD in the #48 family was significantly negatively correlated with chlorophyll but extremely significantly positively correlated with POD and EL.

After heat stress at 43 °C, the POD activity in the #45 family was positively correlated with the five physiological indicators, while that in the #48 family was negatively correlated with the other four indicators except chlorophyll b, which was positively correlated and not significant. At the same time, after heat stress at 43 °C, the EL of the #48 family was negatively correlated with these five physiological parameters.

## 4 Discussion

The cell membrane constitutes the primary heat stress target in plants. The lipid molecules on the biofilm transform from a colloidal state to a liquid crystal state under thermal stress, which increases the permeability of the lipid membrane and allows the penetration of intracellular electrolytes, which leads to an increase in EL (Lastra et al., 1982). There is a positive correlation between the rate of lipid peroxidation and EL: with increasing heat stress, the relative permeability of the plasma membrane and EL also increases (Lukatkin, 2003). In the present study, the EL of each family of *C. fortunei* gradually increased with the intensification of heat stress and exhibited an overall “S”-shaped curve. At 25°C-37°C, the EL increased slowly and then increased sharply after 37°C. The EL of *C. fortunei* family #48 was at a lower level during the whole stress process, indicating that #48 had less electrolyte extravasation, less damage to the cell membrane, and stronger heat resistance.

We found that heat stress led to a decrease in photosynthetic pigment content and impaired chlorophyll biosynthesis in plastids (Marchand et al., 2005; Dutta et al., 2009). Chlorophyll content can reflect the degree of heat stress of plants to a certain extent, thus reflecting the heat tolerance of plants (Bhusal et al., 2018). Studies have shown that heat stress leads to increased decomposition of chlorophyll in plants. Part of the reason may be that high temperature inhibits chlorophyll biosynthesis, resulting in a lower synthesis rate than the decomposition rate. Another reason may be that the content of reactive oxygen species in plants increases under heat stress, thus causing oxidative damage, which then accelerates the degradation of chlorophyll (Sun et al., 2006). The chlorophyll a and chlorophyll b contents in #45 after heat stress (43°C) decreased by 44.4% and 64.6%, respectively, compared with those of the control, while the contents of chlorophyll a and chlorophyll b in #48 decreased by 24.8% and 49.6% respectively, indicating that chlorophyll b in *C. fortunei* was more sensitive to heat stress than chlorophyll a. With the intensification of heat stress, the chlorophyll a content in the cedar family showed a downward trend, and the chlorophyll b and chlorophyll (a+b) contents changed in the same way. However, chlorophyll contents of #45 and #48 increased at 37 °C and 39 °C respectively. Therefore, we speculate that after adapting to high-temperature stress, *C. fortunei* scavenges reactive oxygen species to alleviate high-temperature damage through the initiation of various anti-high-temperature mechanisms, thus leading to the restoration of the chlorophyll content (Chang, 2013). The decrease in chlorophyll content at the beginning of stress may be due to the sudden high temperature affecting the normal physiological state of *C. fortunei* cells, and the accumulation of intracellular reactive oxygen species, which inhibits the biosynthesis of chlorophyll in cells and accelerates the decomposition of chlorophyll.

The enzyme reaction system formed during the long-term evolution of plants. To eliminate oxidative stress caused by heat stress and enhance plant protection, SOD and POD are key enzymes in the plant defense system (Larkindale and Vierling, 2008). These enzymes exist in various plant cells and can scavenge intracellular O^2-^, balance oxygen free radicals, and maintain their metabolic stability (Vidya et al., 2018). In our study, the decrease in SOD activity in #45 at 33°C may be due to the sudden high temperature disrupting the normal physiological metabolism of cedar trees, whereas the subsequent increase in activity is due to a self-protective response to a stressful environment after thermos table exercise. The changes in POD activity in the two cedar families in this study were similar to those in SOD activity, showing a trend of first increasing and then decreasing. Chloroplasts are believed to be the most sensitive organelles (Lütz et al., 2012). Without heat stress, the SOD activity of #48 was higher than that of #45. After heat stress at 39°C, the SOD activity of #48 was significantly higher than that of #45, indicating that #48 could more effectively remove ROS. In addition, correlation analysis showed that under 43°C heat stress, the SOD activity and chlorophyll content of #48 were significantly positively correlated, while the chlorophyll content of #45 during this period was not significantly negatively correlated with SOD activity. Free radicals may cause damage to the chloroplast membrane in plants (Zhang et al., 2021). This result indicated that the increase in the active oxygen content in plants from the #45 family under heat stress caused more serious oxidative damage, which accelerated the degradation of chlorophyll. This further illustrated that #45 has a lower heat resistance than #48.

We found that in the process of heat stress, the chloroplasts of plant leaves expanded and deformed, the thylakoid sheets were loose, the structure was disordered, the plastid globules increased, and the chloroplast envelope was ruptured and disintegrated. Among them, photosystem II on the thylakoid membrane was one of the most sensitive parts, and the damage from heat stress was irreversible (Sheng et al., 2006; Guo et al., 2018). In this study, with the intensification of heat stress, osmiophilic granules increased in the chloroplasts of both families, and the chloroplasts in the needles of the two families were damaged to different degrees under heat stress. Studies have shown that osmiophilic granules are the product of degradation of thylakoid membrane lipid aggregates, which reflects the increased degree of chloroplast damage under stress conditions (Zhou et al., 2018).

The expression of the APX gene was induced by high temperature, drought, low temperature, salt and other stresses. The product of this gene can efficiently remove H_2_O_2_ in plants, thereby maintaining cellular redox homeostasis and improving plant stress resistance (Krasensky and Jonak, 2012). Using wild-type and mutant Arabidopsis plants as materials, the results showed that *APX1*, *APX2* and *APX6* were significantly induced by heat stress, and *APX3* and *APX5* showed obvious inducible expression in response to heat stress (Li et al., 2019). The accumulation of HSPs is controlled by HSFs and plays a central role in the plant heat shock response and the acquisition of heat tolerance by plants or other organisms (Tian et al., 2021). For example, *AtHSFa* and *AtHSFb* in the Arabidopsis A1 subfamily can regulate the expression of related genes and the synthesis of HSP genes in the early stage of the plant response to heat stress, thus helping the plant resist to the damage caused by high temperature (Baniwal et al., 2004; Ikeda et al., 2011). In this study, during heat stress, the expression of each HSF gene in the #48 family was higher than that in the #45 family after 9 h of treatment. In general, the expression of heat resistance genes in the #48 family was higher under heat stress, which explained why the #48 family was more heat-tolerant than the #45 family at the molecular level, and the results were also consistent with previous research results.

## 5 Conclusions

We found that the LT_50_ values of #45 and #48 were the lowest at 39.9°C and the highest at 43.1°C, respectively, out of the eight *C. fortunei* families, which indicated that #45 had the weakest heat resistance and #48 had the strongest heat resistance. In addition, the chlorophyll content and the antioxidant system were closely related to heat tolerance. At the same time, we found that changes in mesophyll cell ultrastructure and chloroplast cross-sectional area were closely related heat stress parameters in *C. fortunei*. Heat stress also induced up-regulation of the *CfAPX1*, *CfAPX2*, *CfHSP11*, *CfHSP21*, *CfHSP70*, *CfHSFA1a*, *CfHSFB2a* and *CfHSFB4* genes, and each gene was significantly different under different heat stress treatments in families #45 and #48. This study lays a foundation for studying the molecular mechanisms of the response and tolerance of *C. fortunei* to heat stress.

## Data availability statement

The data presented in the study are deposited in the NCBI repository, accession number PRJNA644276.

## Author contributions

JYX: Methodology, Validation, Writing-original draft. PZ: Methodology, Validation. JX: Conceptualization, Writing-review & editing, Funding acquisition project administration. JC: Methodology, Validation, Data curation, Writing-original draft, Resources. YZ: Methodology, Validation, Data curation. JY: Data curation, Resources. LZ: Validation, Data curation. HH: Validation, Data curation. All authors contributed to the article and approved the submitted version.
